# *Piriformospora indica* promotes early flowering in *Arabidopsis* through regulation of the photoperiod and gibberellin pathways

**DOI:** 10.1371/journal.pone.0189791

**Published:** 2017-12-19

**Authors:** Rui Pan, Le Xu, Qiao Wei, Chu Wu, Wenlin Tang, Ralf Oelmüller, Wenying Zhang

**Affiliations:** 1 Hubei Collaborative Innovation Center for Grain Industry/ Hubei Key Laboratory of Waterlogging Disaster and Agricultural Use of Wetland, Yangtze University, Jingzhou, China; 2 College of Horticulture and Gardening, Yangtze University, Jingzhou, China; 3 Friedrich-Schiller-University Jena, Institute of General Botany and Plant Physiology, Jena, Germany; University of Tasmania, AUSTRALIA

## Abstract

Flowering in plants is synchronized by both environmental cues and internal regulatory factors. Previous studies have shown that the endophytic fungus *Piriformospora indica* promotes the growth and early flowering in *Coleus forskohlii* (a medicinal plant) and *Arabidopsis*. To further dissect the impact of *P*. *indica* on pathways responsible for flowering time in *Arabidopsis*, we co-cultivated *Arabidopsis* with *P*. *indica* and used RT-qPCR to analyze the main gene regulation networks involved in flowering. Our results revealed that the symbiotic interaction of *Arabidopsis* with *P*. *indica* promotes early flower development and the number of siliques. In addition, expression of the core flowering regulatory gene *FLOWERING LOCUS T* (*FT*), of genes controlling the photoperiod [*CRYPTOCHROMES* (*CRY1*, *CRY2*) and *PHYTOCHROME B* (*PHYB*)] and those related to gibberellin (GA) functions (*RGA1*, *AGL24*, *GA3*, and *MYB5*) were induced by the fungus, while key genes controlling the age and autonomous pathways remained unchanged. Moreover, early flowering promotion conferred by *P*. *indica* was promoted by exogenous GA and inhabited by GA inhibitor, and this effect could be observed under long day and neutral day photoperiod. Therefore, our data suggested that *P*. *indica* promotes early flowering in *Arabidopsis* likely through photoperiod and GA rather than age or the autonomous pathway.

## Introduction

The root endophyte fungus *P*. *indica* is a basidiomycete of the Sebacinaceae family and mimics arbuscular mycorrhizal fungi (AMF) in many aspects[1]. *P*. *indica* can colonize the roots of a broad range of hosts including monocot and dicot plants. It enhances the tolerance of colonized plants against drought, acidity, heavy metals, and various other abiotic stresses as well as biotic stresses[1–8]. In contrast to AMF, *P*. *indica* can be easily cultivated in axenic culture, and it could be very useful for crop improvement and sustainable agriculture[4].

Flowering set the switch from the vegetative to reproductive development and is a prerequisite for crop production. Late flowering cause long generation times and always severely hampers breeding success. Thus, research on flowering time regulation is important for genetic improvement in crops[9].

Regulation of flowering time in the model plant *Arabidopsis* is tightly controlled by various endogenous and environmental cues and has been extensively studied, and the central flowering-time regulatory pathways are conserved across the plant kingdom in general.

Various input signals activate signal transduction pathways which control flowering time. Among them are genes required for controlling the photoperiod, the vernalization time (long exposure to cold), gibberellin (GA) biosynthesis and signaling, age pathway as well as the autonomous (genetic makeup) pathway[10,11]. The photoperiod pathway refers to response to day length and quality of light and it plays a crucial role in controlling flowering in *Arabidopsis*[11,12]. According to an external coincidence model, light resets the circadian clock which controls proper oscillation of the *CONSTANTS* (*CO*) mRNA level, and also regulates the CO protein stability[12].

Far-red and blue light promote flowering through phytochrome A (PHYA) and cryptochromes (cryptochromes 1 and cryptochromes 2, CRY1 and CRY2), respectively. Red light inhibits flowering through PHYB function across a range of species. *PHYA*, *CRY1*, and *CRY2* have been shown to prevent CO protein degradation, whereas *PHYB* promotes its degradation[13]. The increase in the CO protein level activates the transcription of *FLOWERING LOCUS T* (*FT*) only under long day conditions. FT then moves through the phloem to the meristem, where it associates with FLOWERING LOCUS D (FD), and the FT-FD complex promotes expression of the *SUPPRESSOR OF OVEREXPRESSION OF CONSTANS1* (*SOC1*) gene and downstream floral meristem identity genes, such as *APETALA1* (*AP1*) and *LEAFY* (*LFY*) to induce flowering[14–17].

The vernalization pathway accelerates flowering on exposure to a longer cold periods through the floral repressor *FLOWERING LOCUS C* (*FLC*) and other *FLC* clade members. After vernalization, *FLC* expression is strongly repressed through histone modifications which leads to the activation of the downstream floral integrator genes *FT* and *SOC1* to promote flowering [18].

In *Arabidopsis*, GA signaling is required for floral induction. GA directly promotes *SOC1* and *LFY* expression and the increased level of *SOC1 mRNA*, which, in turn, activates the downstream genes *LFY* and *AP1* to induce flowering. This relay of information from GA to *SOC1* occurs dependent of DELLA proteins degradation such as the RGA, RGL2 and with a partial contribution of RGL1[19]. Kim et al. [20] reported that *P*. *indica-*inoculated *Arabidopsis* plants displayed a significant early flowering phenotype, activation of flowering regulatory and GA biosynthetic genes, and an increase in GA4 content. Their data indicated that the GA pathway in *Arabidopsis* is targeted by *P*. *indica* and might be responsible for the early flowering effect.

The autonomous pathway function normally to limit the accumulation of *FLC* expression level throughout development stages independent of the photoperiod and GA[21]. Age pathway involved in flowering regulation independent of photoperiod vernalization, and GA pathway. MicroRNA and SPL (SQUAMOSA PROMOTER BINDING PROTEIN-LIKE) are key components in age pathway[22]. Taken together, these core pathways regulate the expression of central floral pathway integrators, such as *FT* and *SOC1* which in turn regulate the downstream floral identity genes to control flowering[11, 12].

Although early flowering in *Arabidopsis* was induced by *P*. *indica* through activating the GA pathway and regulating the expression level of floral integrators[20], other factors regulating the flowering time are less investigated. Our results confirm previous observations that *P*. *indica* promotes flower development and silique production. We show that the core flowering regulatory gene *FLOWERING LOCUS T* (*FT*), the photoreceptor genes controlling the photoperiod (*CRY1* and *PHYB*), as well as the GA-related genes *REPRESSOR OF ga1-3* (*RGA1*), *AGAMOUSLIKE 24* (*AGL24*), *GA3* (*GA REQUIRING 3*), and the bHLH transcription factor *MYB5* are up-regulated by *P*. *indica*, while the key genes for the age and autonomous pathways remained unchanged. Early flowering promotion effect can be observed under long day and neutral day photoperiod. Therefore, our data indicate that *P*. *indica* promotes early flowering in *Arabidopsis* likely through photoperiod and the GA pathway rather than age or the autonomous pathway.

## Results

### Plant growth, flowering time

**Fig 1:**
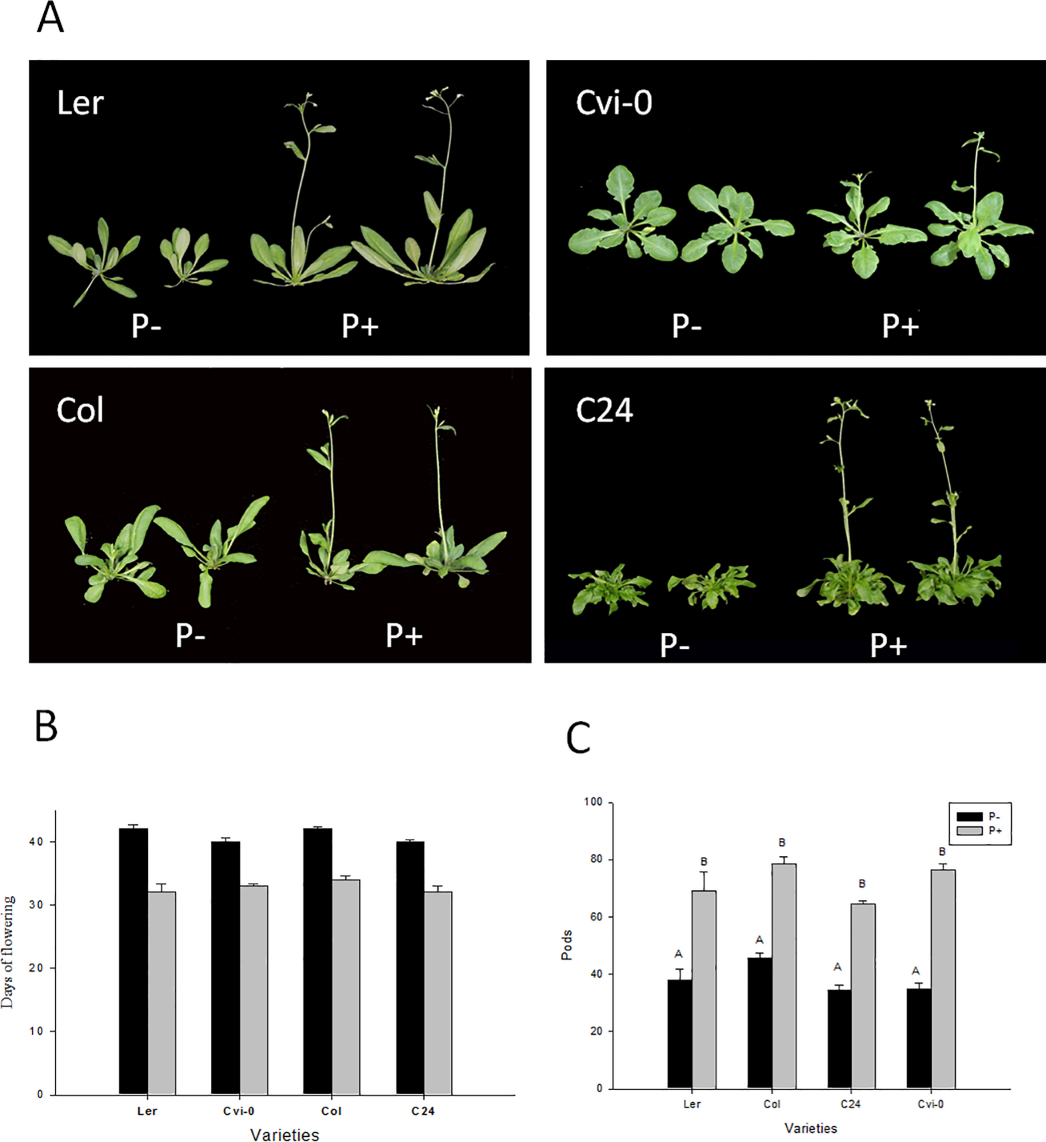
Influence of *P*. *indica* on flowering of *Arabidopsis* and silique numbers grown in soil. (A) Picture of 5-week-old *Arabidopsis* (4 ecotypes) plants with (+P, right) or without (-P, left) *P*. *indica* inoculation. (B) Days until flowering. (C) Silique number of *P*. *indica*-colonized and non-colonized plants. Experiments were repeated independent for four times with similar results. “*” indicates significant difference (p<0.05) according to Student’s *t*-test.

**Fig 2:**
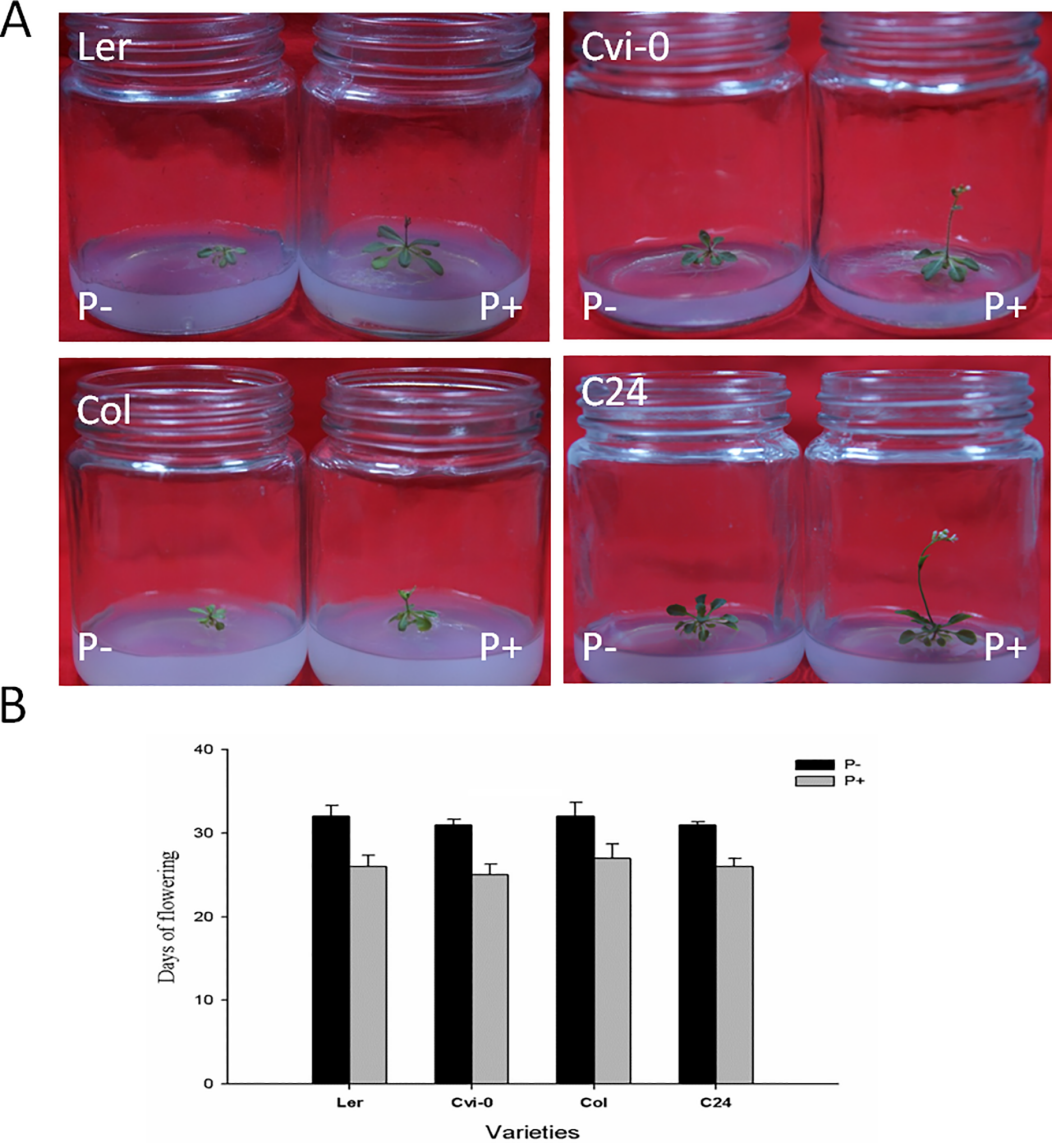
Influence of *P*. *indica* on flowering in *Arabidopsis* grown on sterile culture. Picture (A) and flowering time (B) of 4-week *Arabidopsis* (4 ecotypes) plants with (+P, right) or without (-P, left) *P*. *indica* inoculation in sterile MS medium. Experiments repeated for four times and each replicate represents 18 samples. “*” indicates significant difference at p<0.05 according to Student’s *t*-test.

Microscopic inspection of the roots of *Arabidopsis* colonized by *P*. *indica* to ensure that the fungus enters roots and grows intracellularly in the root cortex (S1 Fig). Under our experimental conditions, the heights and leaf areas of *P*. *indica-*colonized plants were significantly increased compared to non-colonized plants (Figs 1A and 2A). Also the number of siliques produced by four investigated *Arabidopsis* ecotypes Ler, Col, C24, and Cvi-0 was ~2-fold higher in the presence of the fungus (Fig 1C).

We observed that Ler, Cvi-0, Col and C24 plants grown in soil flowered on the 32^nd^ day, 34^th^ day, 33^rd^, and 32^nd^ day, respectively. *P*. *indica* inoculation promotes earlier flowering of Ler, Cvi-0, Col and C24 plants for 10 days, 7 days, 8 days, and 8 days, respectively (Figs 1 and 2). This demonstrated that the early flowering induced by *P*. *indica* is ecotype independent. Similar results were obtained for plants grown in sterile cultures, and we observed that Ler, Col, Cvi-0, and C24 plants inoculated by *P*. *indica* flowered 5 to 6 days earlier than those grown without *P*. *indica* inoculation (Fig 2). Apparently, the stimulatory effect of the fungus on flowering time is independent of the growth conditions and ecotype.

### *P*. *indica* affects the expression of flowering-regulatory genes

To determine the pathway by which *P*. *indica* induces early flowering, we checked the expression levels of key genes in the photoperiod, GA, autonomous, vernalization and age pathways between 8 to 13 days after inoculation (DAI).

**Fig 3:**
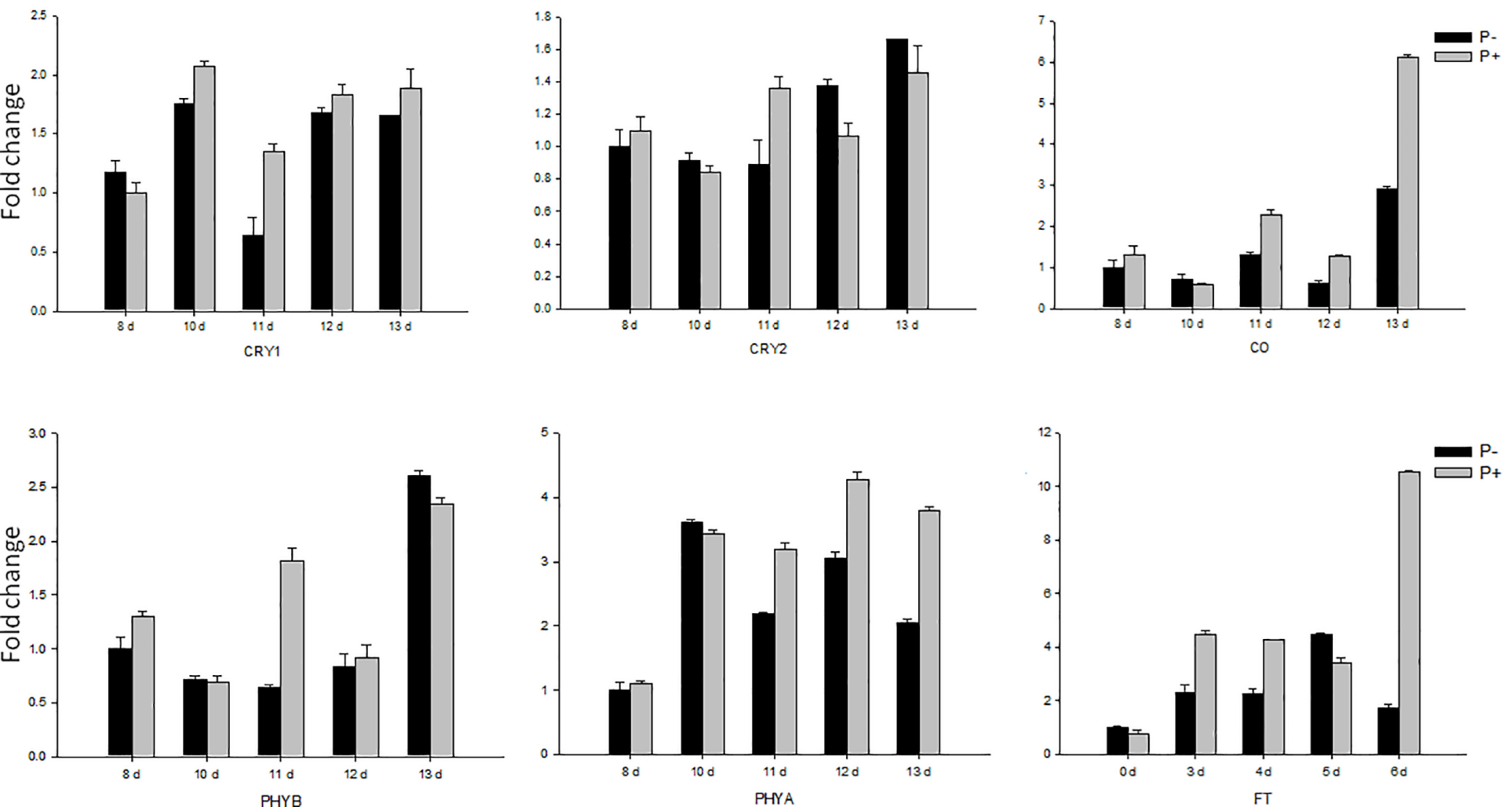
Gene expression of key genes in photoperiod pathway responsible for flowering. Col in sterile culture was chosen for gene expression. The samples were harvested 8, 10, 11, 12 and 13 DAI for RT-qPCR. Bars represent SEs of three technical repeates. Experiments were repeated independent for three times with similar results and one representative result was showed.

Phytochromes mainly absorb red and far-red light and phytochrome A (*PHYA*) promotes flowering while *PHYB* inhibits flowering. The mRNA level of *PHYA* is 1.5–2 times higher in *P*. *indica-*colonized *Arabidopsis* (11, 12 and 13 DAI) compared to uncolonized plants, while that of *PHYB* showed no significant difference between plants with *P*. *indica* inoculation and without. The blue/ultraviolet-absorbing cryptochromes *CRY1* and *CRY2* promote flowering. The mRNA level of *CRY1* in *P*. *indica-*inoculated *Arabidopsis* is 2.1 times higher than in the uncolonized control 8 DAI while the mRNA level of *CRY2* showed no significant difference. *CO*, a key gene in the signal output way of the biological clock is regulated by the clock and generates 24 hours of periodic oscillation[10]. Fig 3 demonstrates that the periodicity of the *CO* mRNA level in *P*. *indica-*inoculated *Arabidopsis* is impaired and the mRNA level is higher in *P*. *indica-*inoculated plants compared to the uncolonized controls between 7 and 10 DAI. In particular, the strong increase in the *CO* mRNA level during the later time points may participate in early flowering. The *FT* mRNA level increased even stronger in the presence of *P*. *indica*. *FT* expression is activated by CO, which is a signal for photoperiodic signaling transported from leaf to shoot meristem to induce floral development, and high expression level of *FT* promote flowering (Fig 3).

**Fig 4:**
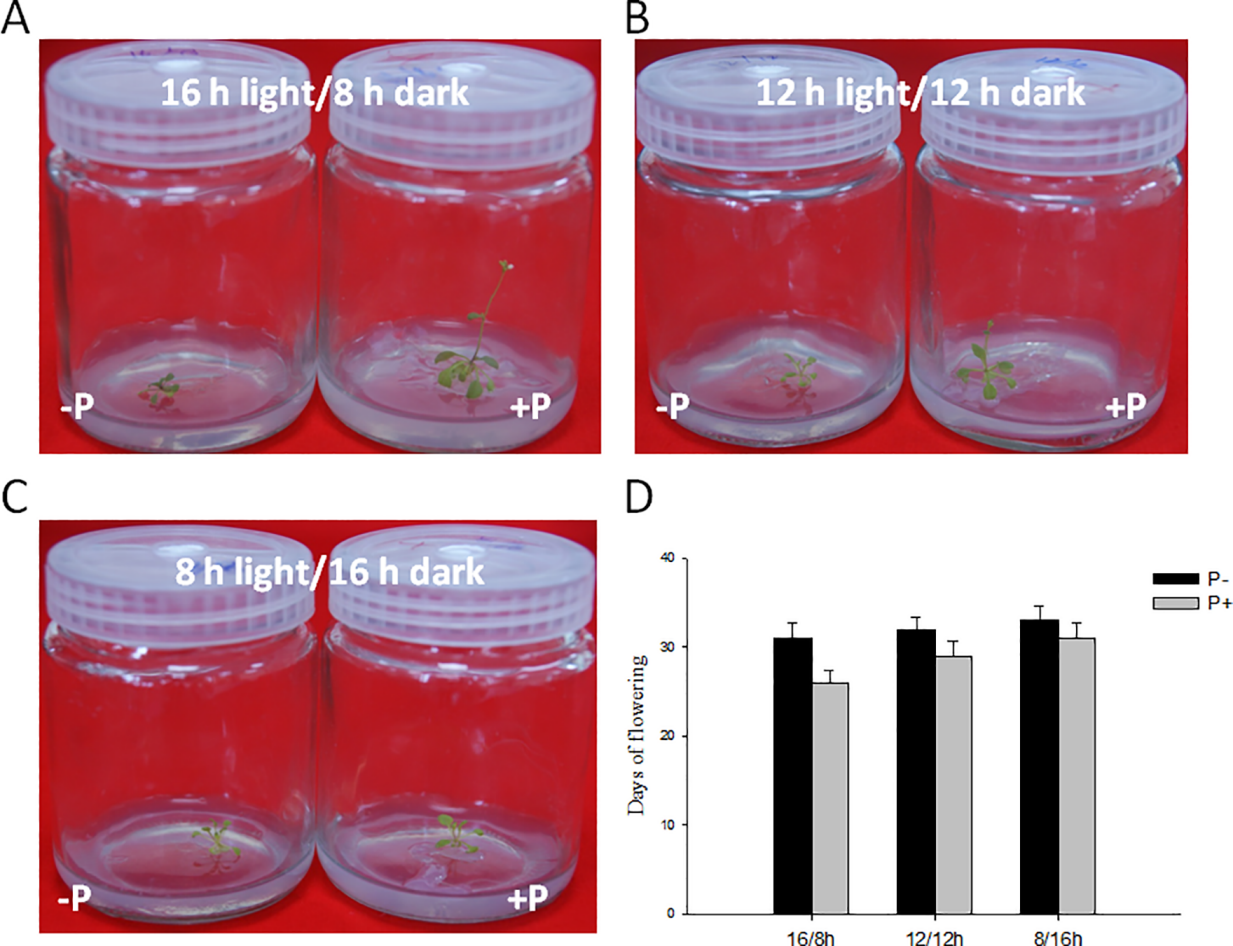
Phenotype of Col plants colonized with or without *P*.*indica* under long day, neutral day, and short day conditions for 14 days.

Given that genes of the photoperiod pathway responsible for flowering time responded to the fungal treatment, we placed the plants under long day, neutral day, and short day photoperiod conditions. We observed that plants grown under long day conditions flowered (the 31^**st**^ day) a little earlier than those grown under neutral day (the 32^**nd**^ day), and short day photoperiod conditions (the 33^**rd**^ day). *P*. *indica* inoculation promotes flowering of *Arabidopsis* under long, neutral, and short day conditions for 5 days, 3 days, and 1 day, respectively (Fig 4).

**Fig 5:**
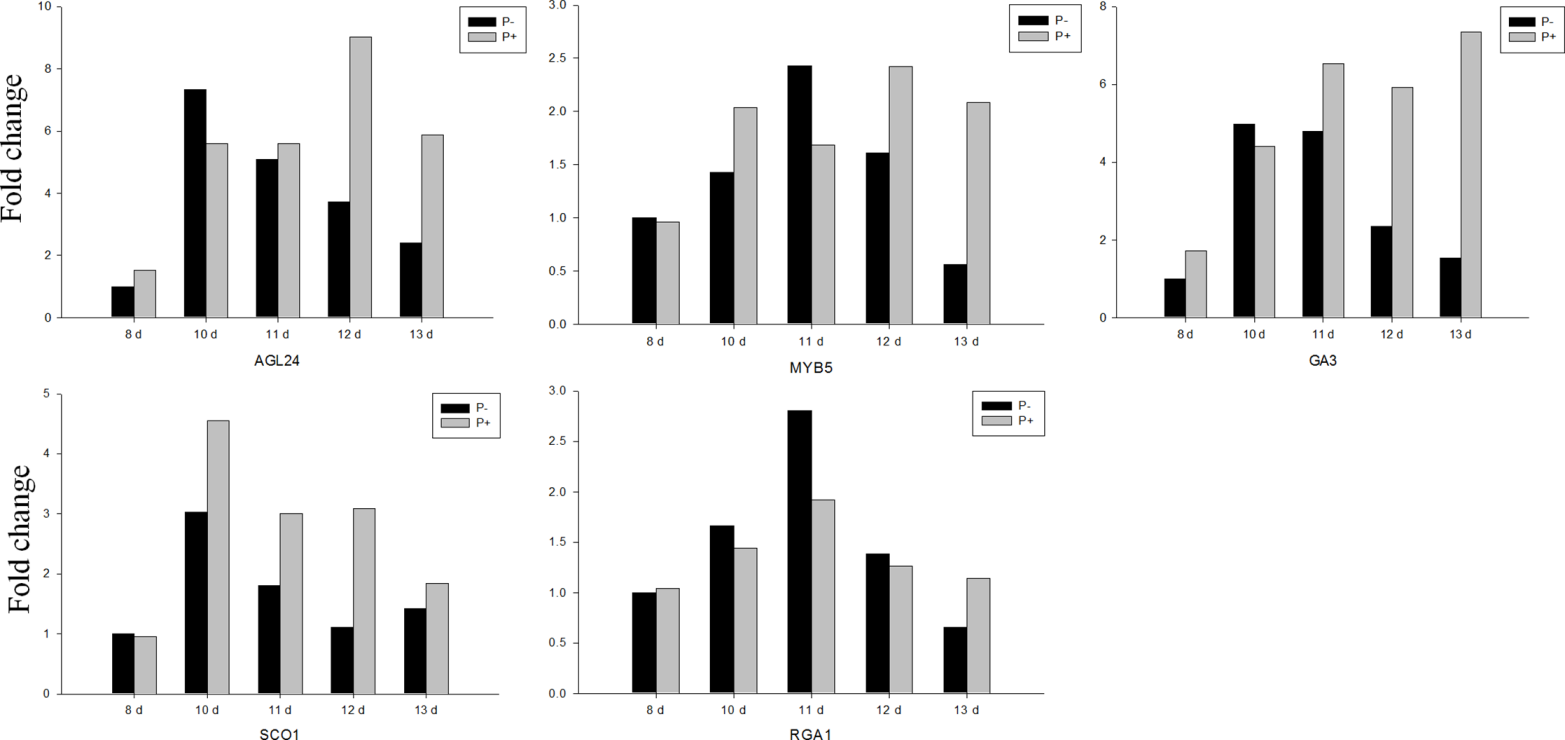
Gene expression of key genes in GA pathway responsible for flowering. Col in sterile culture was chosen for gene expression. The samples were harvested at 8, 10, 11, 12 and 13 DAI for RT-qPCR. Bars represent SEs of three technical repeats. Experiments were repeated independent for three times with similar results and one representative result was showed.

GAs act as major factors promoting flowering in *Arabidopsis*. The *ENT-KAURENE OXIDASE 1* (*GA-REQUIRING3*, *GA3*) gene encodes a member of the cytochrome P450 protein family that is involved in later steps of the gibberellin biosynthetic pathway. The expression level of *GA3* in *P*. *indica-*inoculated *Arabidopsis* increased during the co-cultivation experiment and was ~ 5-times higher in the presence of the fungus after 13 days of co-cultivation. *RGA1*, which belongs to the DELLA gene family, negatively regulates flowering, and the expression level of *RGA1* was slightly reduced in plants colonized by *P*.*indica* for 10 and 11 days. *MiR159* is regulated by DELLA protein, and *MYB5* also responded to *P*. *indica* treatment which function downstream of *miR159*. *SCO1* is the key gene regulating flowering in response to GA. The mRNA level of *SCO1* responded to *P*. *indica as* well. Finally, the stimulatory effect of the fungus on the mRNA level of the GA responsive gene *AGAMOUSLIKE 24* (*AGL24*) supports the idea that this phytohormone might be a target of the fungus (Fig 5).

**Fig 6:**
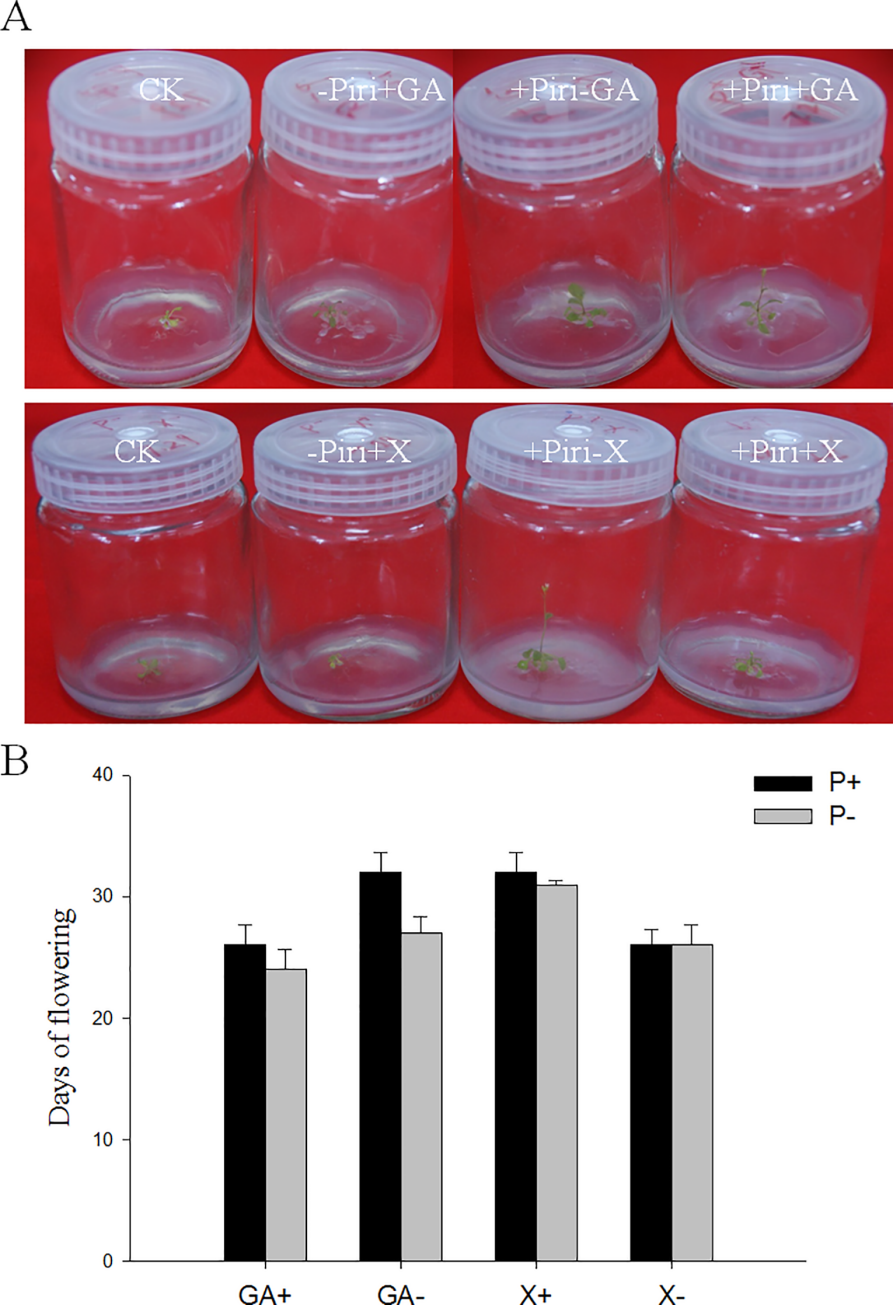
Effect of supplementation with GA, and uniconazole in MS medium on the flowering phenotype of *P*. *indica*-colonized *Arabidopsis* plants. 0.1 mM GA and 10 μM GA inhibitor uniconazole was added to the MS medium. Bars represent SEs and are based on 3 independent experiments.

Given that genes in the GA pathway, which control flowering time, were influenced significantly by the fungus, we treated the wild-type plants with 0.1 mM GA and 10 μM GA inhibitor uniconazole before transferring to normal growth conditions. GA promoted both growth and flowering of Col plants in the presence and absence of *P*. *indica*. In contrast, the early-flowering phenotype conferred by *P*. *indica* was vanished by the treatment with uniconazole (Fig 6). These results indicated that the GA pathway is at least partially responsible for *P*. *indica*-induced early-flowering.

**Fig 7:**
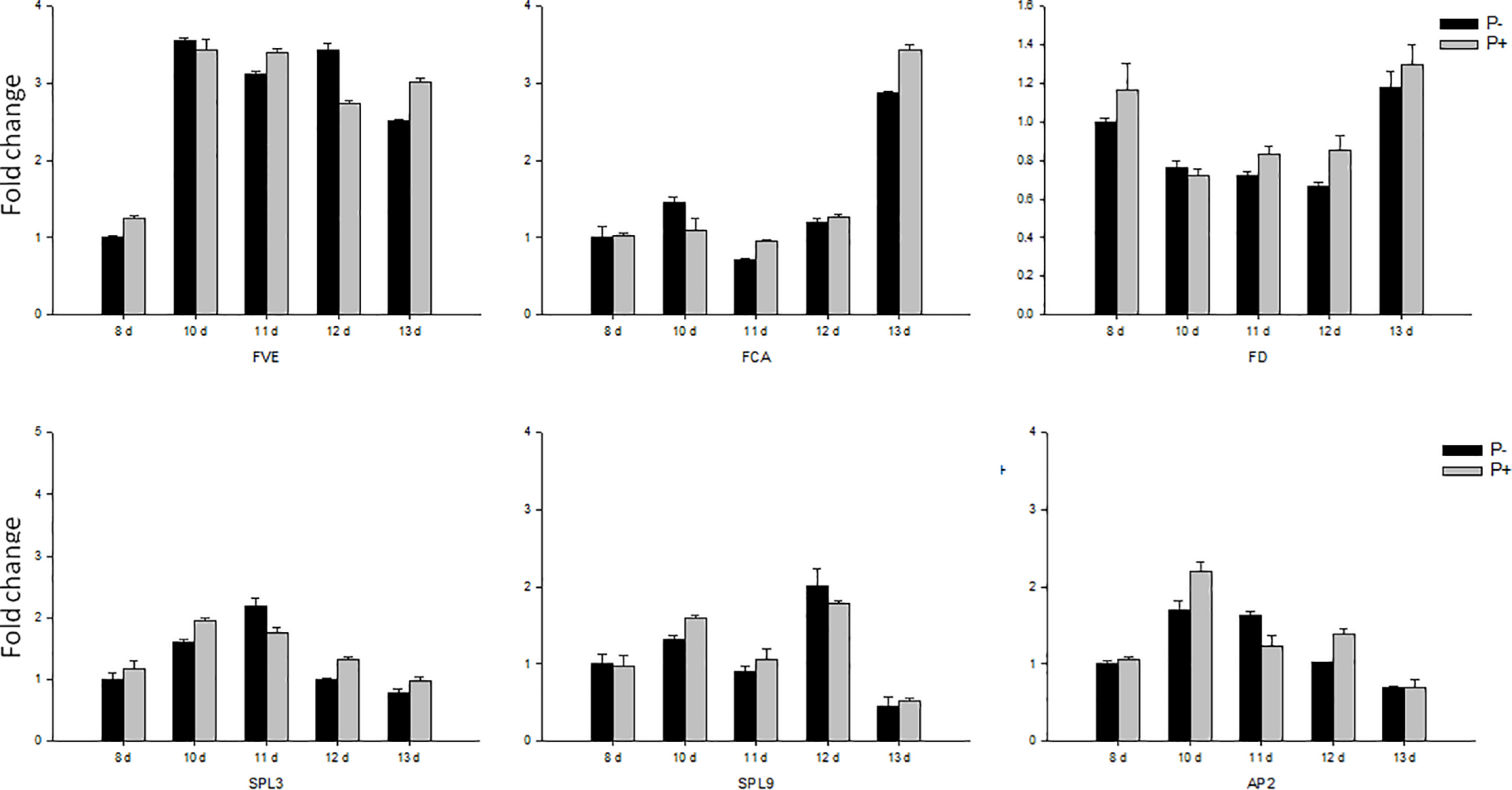
Gene expression of key genes in the autonomous and age pathways responsible for flowering. Col in sterile culture was chosen for gene expression analyses. The samples were harvested at 8 DAI, 10 DAI, 11 DAI, 12 DAI, and 13 DAI for RT-qPCR. Bars represent SEs of three technical repeats. Experiments were repeated independent for three times with similar results and one representative result was showed.

We also checked the response of key genes involved in other flowering controlling pathways. *FVE* and *FCA*, two representatives of the autonomous pathway, showed no significant response to *P*. *indica* and the expression of *FD* which codes for an enzyme located downstream of FVE and FCA, also did not respond to the fungus (Fig 7). Thus, the autonomous pathway appears to be not a target of *P*. *indica* in controlling flowering.

**Fig 8:**
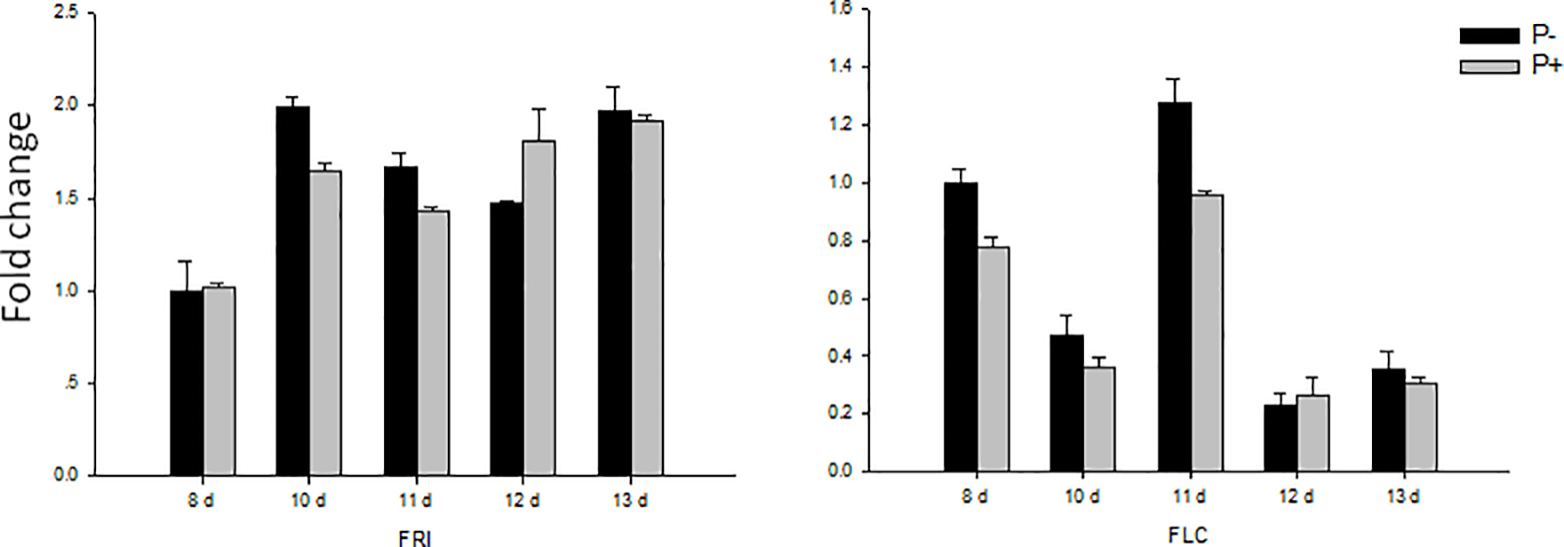
Gene expression of key genes in vernalization pathway responsible for flowering. Col in sterile culture was chosen for gene expression. The samples were harvested at 8, 10, 11, 12 and 13 DAI for RT-qPCR. Bars represent SEs of three technical repeats. Experiments were repeated independent for three times with similar results and one representative result was showed.

*SQUAMOSA PROMOTER BINDING PROTEIN-LIKE 3 and -9* (*SPL3*, *-9*) are the key genes of the age pathway, and they are responsible for flower initiation during aging. The *SPL3* and *SPL9* transcript levels were comparable in colonized and uncolonized plants, and a downstream gene of SPL3 and -9, *APEATLA2* (*AP2*) also showed no stimulation after *P*. *indica* inoculation. Finally, both the *FLOWERING LOCUS C* (*FLC*), and the gene for its activator, *FRI* (*FRIGIDA*), are key components of the vernalization pathway, and both genes did not respond to *P*. *indica* as well (Fig 8).

These results strongly suggest that the GA and light-dependent pathways are targets of *P*. *indica* to promote early flowering rather than the autonomous and age pathways.

## Discussion

In the past, early flowering is often observed in stressful environments as a compensatory mechanism which enables the plant to efficiently use its resources to produce at least a few viable seeds[23,24]. However, in our study, we found *P*.*indica* promotes both the flowering and siliques production of *Arabidopsis*. In recent years, early flowering is more considered as an effective way to shorten the growth period and thus increases yield. Early flowering can be utilized for high-speed breeding and helps plant to avoid abiotic hazards, such as high temperature or chilling damage, especially in regions with four distinctive seasons. In addition, for some medical and ornamental plants, early flowering has important economic advantages.

In the present studies, *P*. *indica* was axenically cultivated and applied to the *Arabidopsis* grown in soil or medium in order to analyze its effects on the expression of genes representing the four main pathways controlling flowering time. We observed that *P*. *indica* promotes early flowering, similar to findings reported by others for *Arabidopsis* and other host species [25–27].

A possible explanation for the faster flower development induced by *P*. *indica* could be earlier expression of developmentally regulated and flower controlling genes [20]. Two photoreceptor families, *PHYA*, *PHYB* and *CRY1*, *CRY2*, control the light cycle. The periodic signal is transmitted to the biological clock, which regulates expression of the key gene *CO*. In our investigation, the expression level of *PHYA* and *CRY1* are higher in *P*. *indica-*colonized plants while *PHYB* and *CRY2* remain unchanged. Moreover, early flowering promotion conferred by *P*. *indica* was photoperiod dependent (Fig 4). Thus, we suggested that the photoperiod pathway was an effective channel influenced by *P*. *indica* to promote early flowering.

In our study, several key genes in the GA pathway responsible for the control of flowering time were influenced significantly by the fungus, which is consistent with the observation that early flower induction is GA dependent[20]. In addition, levels of gibberellins in *P*. *indica* colonized roots increased in Chinese cabbage and barley seedlings and a GA2ox gene mediating inactivation of active GA was found to be down-regulated in barley plants in response to P. indica inoculation, suggesting that GA biosynthesis is enhanced by *P*. *indica* [28–31]. An up-regulation of gibberellin biosynthetic (Gibberellin 20-Oxidase2, Gibberellin 3-Oxidase1 and Gibberellin requiring 1) genes and an increase in GA4 content were found in *P*. *indica* colonized plants and these data indicate that *P*. *indica* promotes early flowering in *Arabidopsis* likely by increasing gibberellin content[20]. Therefore, the GA pathway is at least partially responsible for the early-flowering phenotype conferred by *P*. *indica* inoculation.

In a recent study, DELLA was shown to interact with FLC to enhance the inhibition ability of FLC. The inhibition ability of FLC is reduced when exogenous GA promoted the degradation of DELLA [10]. DELLA proteins played key roles in GA and other phytohormones (e.g ethylene) regulated flowering promotion. Several studies revealed that mutants in brassinosteroid (BR) signaling pathway showed a different flowering phenotype[32–35]. BR signaling also interacts with GA and abscisic acid (ABA) pathways signals to control flowering time[36,37]. In addition, ABA, ethylene, and salicylic acid (SA) are also critical in regulating flowering[36, 38, 39].

**Fig 9:**
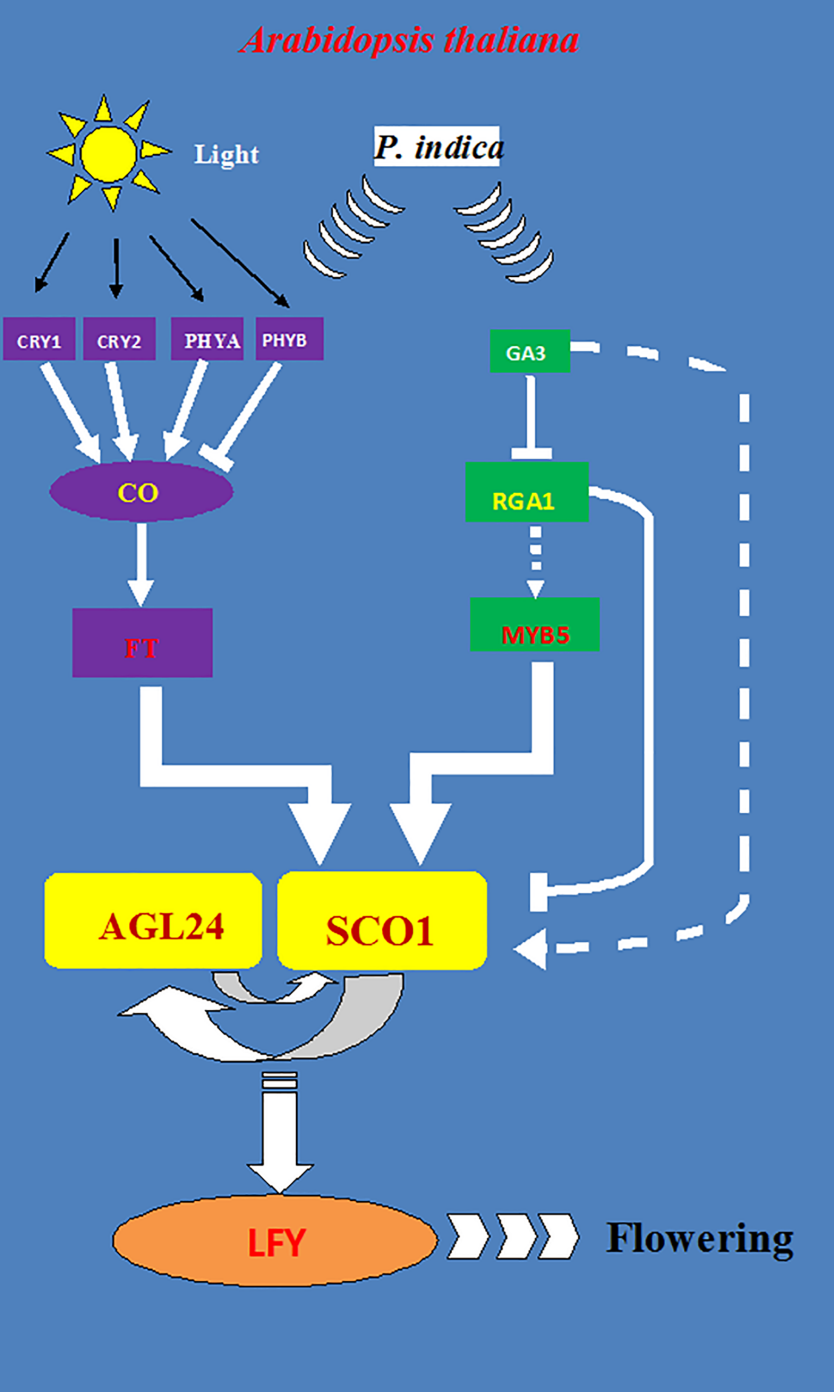
Major flowering pathway genes of *Arabidopsis thaliana* influenced by *P*.*indica* inoculation. Positive and negative regulatory connections are indicated by arrows and lines with T-ends, respectively. Gene name abbreviations are explained in text.

Since we did not observe a stimulation of genes involved in the age and autonomous pathways, we propose that they are not the major targets of the fungus in promoting flower initiation and/or development and most of our work are summarize in Fig 9 using a model according to the review of Hill [40].

Although our results suggested a more specific control of flower initiation and development by *P*. *indica*, the increased growth and thus more efficient uptake of growth-limiting nutrients such as nitrogen and phosphorous could also favor early flowering. *P*. *indica* promotes phosphorous and nitrogen uptake from the soil[41–45]. However, to what extend nutrient availability participates in flower induction is difficult to elucidate because alteration in nutrient supply affects multiple biochemical pathways in plants.

## Materials and methods

### *P*. *indica* growth condition and co-cultivation with *Arabidopsis*

The fungus was cultivated in a 250 mL flask with Aspergillus (ASP) medium. This flask was placed on a shaker (150 r min^-1^), and the fungus was grown in the dark at a constant temperature of 25°C. After 14 days, the liquid was removed by filtration and excess culture medium carefully removed from the mycelia.

Four *Arabidopsis thaliana* ecotypes, Columbia (Col-0), Landsberg (Ler), Cape Verdi Island (Cvi-0), and C24 were used for the flowering experiment in soil, on PMN and MS media. Seeds on agar plates were cold-treated at 4°C for 2 days in the dark. Ten day- old young seedlings (2 plants in a pot and 50 pots were used) were transferred to soil and 1 ml of 0.1% *P*. *indica* suspension were added (1 g fresh mycelium per liter of water, injection of the suspension into the soil), and dead mycelia were added into the soil as a control followed Xu et al. [6]. The growth conditions is 22°C with a 16 h-light and 8 h-dark photoperiod (long day conditions). Ten day- old young seedlings were transferred to PMN medium and co-cultivated with *P*. *indica* for 8 days (4 plants in a petri dish and 20 petri dishes were used for each treatment). Then *Arabidopsis* plants and *P*. *indica* were transferred to bigger glass bottles with MS medium for phenotype observation and gene expression analysis. Short day (8 h-light and 16-h-dark) as well as neutral day (12 h-light and 12 h-dark) were also used to study the effect of the photoperiod on the early flowering phenotype.

Root colonization by *P*. *indica* was examined one week after inoculation. The roots were harvested from each plant and stained with trypan blue. Colonization of roots was assessed using a Leica microscope (DM5000 B; Germany).

### Measurement of flowering time and number of siliques

After checking the colonization of the plants at day 7, they were observed every day for growth promotion and flower induction by photography. For the standardized experiments, *Arabidopsis* seedlings were grown in soil under long day condition (16 h light—8 h dark), 22°C, and flowering time was measured by counting the number of rosette and cauline leaves at the first flower blooming. The experiment was repeated three times and data obtained from 60 plants were statistically analyzed.

### RNA preparation and analysis of gene expression by RT-qPCR

Total RNA was extracted with TRIzol (Invitrogen) according to manufacturer’s instructions. One microgram of total RNA was subjected to first-strand cDNA synthesis using the PrimeScript RT reagent kit with gDNA Eraser (Takara). RT-qPCR was performed by the SYBR green method using the StepOne Plus Real-time PCR system (Applied Biosystems). Real-time quantitative reverse transcription PCR and the 2^-△△Ct^ method[46] was used to calculated the cycle threshold value of each sample. The expression levels of the described key genes were normalized to *ACTIN2*. The experiments were repeated (biological replicates) three times, with similar results, and the representative data from one replicate are shown. Data are means ± SD (n = 3) of three technical replicates. RT-qPCR primers were designed by Primer Primer5 are listed in S1 Table.

### Chemicals supplements

For experiments with GA and uniconazole supplementation, *Arabidopsis* seedlings colonized with *P*. *indica* for 8 days were transferred to MS medium containing 0.1 mM GA (Sigma-Aldrich) according to Domagalska[37] or 10 uM uniconazole (mixture from Sigma-Aldrich). The flowering phenotype was recorded as described above.

## Supporting information

S1 Fig Trypan blue staining of *Arabidopsis* roots showing intracellular *P*. *indica* chlamydospores observed at day 9. Bar, 20 mm.
(TIF)

S1 Table Primers used for RT-qPCR. (DOCX)
