# Supplementary figures and images for: Piriformospora indica promotes early flowering in Arabidopsis through regulation of the photoperiod and gibberellin pathways
Source: PLoS One. 2017 Dec 19;12(12):e0189791. doi: 10.1371/journal.pone.0189791 (PMC5736186; doi:10.1371/journal.pone.0189791)

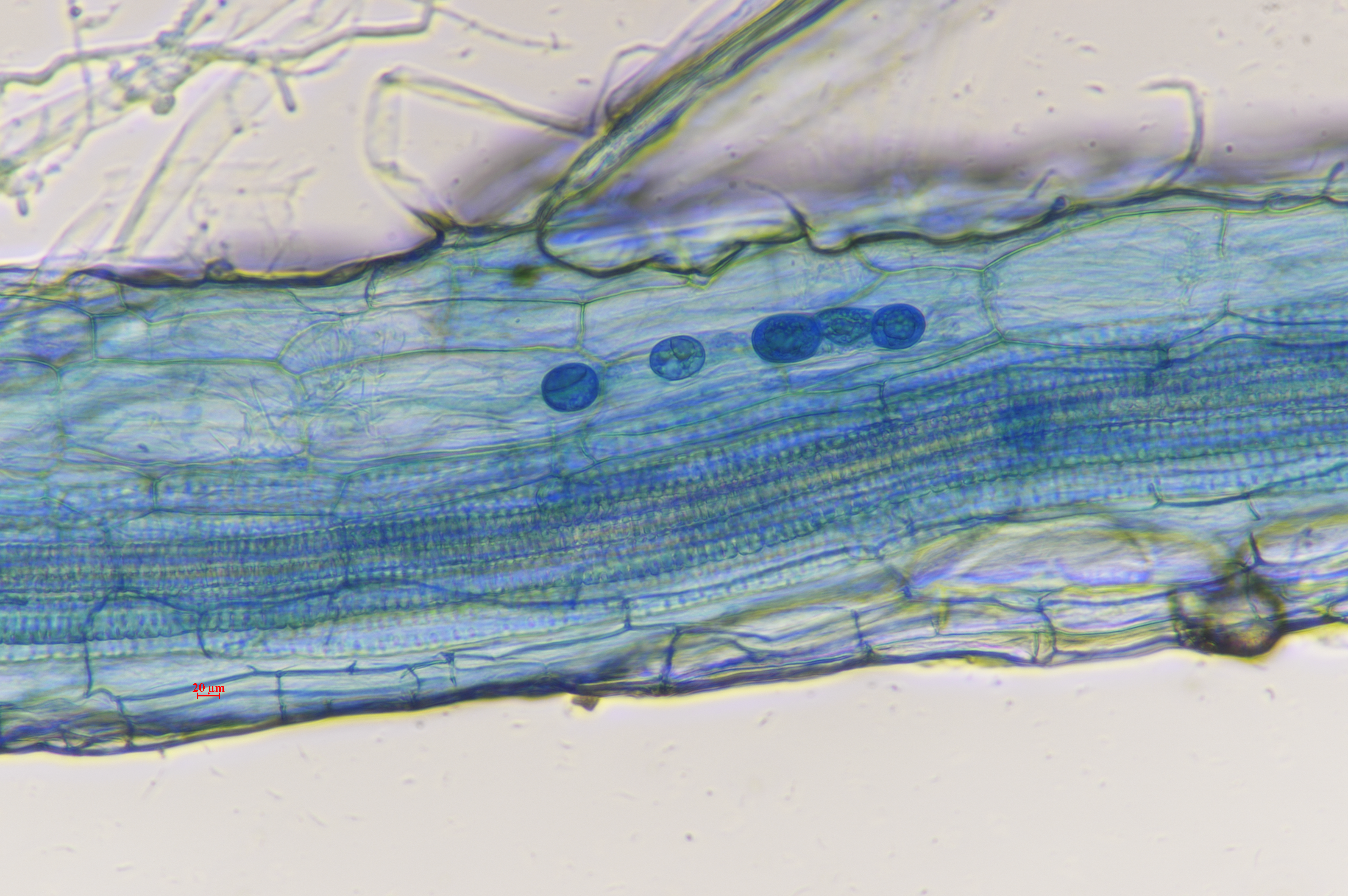

Supplement: S1 Fig — Bar, 20 mm. (TIF) [file pone.0189791.s001.tif]
