# Supplementary material for: Piriformospora indica promotes early flowering in Arabidopsis through regulation of the photoperiod and gibberellin pathways
Source: PLoS One. 2017 Dec 19;12(12):e0189791. doi: 10.1371/journal.pone.0189791 (PMC5736186; doi:10.1371/journal.pone.0189791)
Supplement: S1 Table — (DOCX) [file pone.0189791.s002.docx]

**Supplementary table 1. Primers used for RT-qPCR**

| **gene name** | | **Primer(5'-3')** |
| --- | --- | --- |
| AGL24 | F | GTTCTTTGCGATGCTGATGTTG |
|  | R | GTTTCCGTAGCTGCTTGGTTTT |
| AP2 | F | CAAAGACGCCGTGACCAACT |
|  | R | GGTTCATCCTGAGCCGCAT |
| CO | F | GCTTCGTGGCTGTTCCCTA |
|  | R | CCCGTAGCTCGTCTGTGGT |
| CRY1 | F | AGGTGGTGGCTCAAGAACAG |
|  | R | CTCATAAAGCAAGTCTGCGTTG |
| CRY2 | F | AAACCCACAACACGATTTCAGC |
|  | R | TCCCGAACTAACGAAACAGG |
| FCA | F | TTCACTAAACAAGCAAGCCACTG |
|  | R | TAACAAACCCACATCCACGA |
| FD | F | TCAAACTCTAATCTTCATACCCACC |
|  | R | ATACGCTTATGTCTTCTATTCCCTG |
| FRI | F | AGGAGCCAGCGAAGTTTGT |
|  | R | ATCTTCACCTTCCCTTTACCAC |
| FT | F | CAACCCTCACCTCCGAGAATA |
|  | R | TGCCTGCCAAGCTGTCGA |
| FVE | F | AACCAAACCGTCATGCTGTGC |
|  | R | TGTCCCAATCGTTGTGATGTGA |
| GA3 | F | TACGCAAATCGGAGGCTATCA |
|  | R | TGAAGAGCACCAGCACAAACC |
| MYB5 | F | CTCGGCAACAGGTGGTCATT |
|  | R | CTTCTGGTTTATGGATGTTGTTTGC |
| PHYA | F | TATGCGATTATCCACAGGGTT |
|  | R | ATACTCCCGCTGGGTAAAGAT |
| PHYB | F | AAACCCTCTAATCCCGCCAAT |
|  | R | CTTCACTGCGAGACCAACCC |
| RGA1 | F | ACATCGACTTCGACGGGTACG |
|  | R | AGTCAACCAGGATAACAGAACGAG |
| SCO1 | F | CAAGAGCAAGAACGAGGAATC |
|  | R | GAGGCTCAACACCAGCAACACT |
| SPL3 | F | AAGCCAAACAGTACCACAAACG |
|  | R | TGTGTCCAGCTAAGCGTCTCC |
| SPL9 | F | GGCGACTCAAACTGTGCTCT |
|  | R | CCCATTGCCGTGCCACTACT |
| LFC | F | CGACTTGAACCCAAACCTGA |
|  | R | ACGAGAAGAGCGACGGATG |
